# Adverse Events of Intravesical OnabotulinumtoxinA Injection between Patients with Overactive Bladder and Interstitial Cystitis—Different Mechanisms of Action of Botox on Bladder Dysfunction?

**DOI:** 10.3390/toxins8030075

**Published:** 2016-03-16

**Authors:** Yuh-Chen Kuo, Hann-Chorng Kuo

**Affiliations:** 1 Department of Urology, Yangming Branch of Taipei City Hospital, Taipei 11146, Taiwan; yuhchens@hotmail.com; 2 Department of Urology, Buddhist Tzu Chi General Hospital and Tzu Chi University, Taipei 11146, Taiwan

**Keywords:** onabotulinumtoxinA, adverse events, interstitial cystitis, overactive bladder

## Abstract

Intravesical onabotulinumtoxinA (BoNT-A) injections have been proposed to treat both overactive bladder (OAB) and interstitial cystitis/bladder pain syndrome (IC/BPS) in patients with refractory conditions. We compared adverse events (AEs) after BoNT-A treatment between IC/BPS and OAB in women. IC/BPS patients who failed conventional treatments were enrolled to receive suburothelial injections of BoNT-A (100 U) followed by hydrodistention. Age matched OAB female patients refractory to antimuscarinic agents underwent BoNT-A (100 U) injections. The bladder capacity, maximum flow rate (Qmax), post-void residual (PVR), and voiding efficiency (VE) at baseline, 3 and 6 months, and the post-treatment AEs were analyzed between groups. Finally, 89 IC/BPS and 72 OAB women were included. In the OAB group, the bladder capacity and PVR increased, and VE decreased significantly at three and six months after BoNT-A treatment. In the IC/BPS group, the Qmax increased significantly at six months. There were significant differences in changes of capacity, Qmax, PVR and VE between the two groups. Moreover, OAB patients suffered more frequently from events of hematuria, UTI, and large PVR (>200 mL), but less frequently from events of straining to void. In conclusion, OAB women had higher PVR volume and lower VE than those in IC/BPS after BoNT-A injections. These results imply that the bladder contractility of OAB patients are more susceptible to BoNT-A, which might reflect the different mechanisms of action of Botox on bladder dysfunction. Further investigations to confirm this hypothesis are warranted.

## 1. Introduction

Overactive bladder (OAB) is a symptom syndrome characterized by urinary urgency, usually accompanied by frequency and nocturia, with or without urgency urinary incontinence, in the absence of a urinary tract infection (UTI) or other obvious pathology [1]. OAB symptoms can be quite bothersome and can negatively affect health-related quality of life (HR-QoL), increase anxiety and depression, and increase healthcare usage [2,3]. The cause of OAB is unclear, and indeed there may be multiple causes [4]. It is often associated with overactivity of the detrusor muscle, a pattern of bladder muscle contraction observed during urodynamics, which may be neurogenic, myogenic, urotheliogenic or idiopathic in origin [5].

Intravesical injection of onabotulinumtoxinA (BoNT-A) is approved by the Food and Drug Administration for treatment of OAB refractory to antimuscarinic agents. Several randomized placebo-controlled studies have demonstrated that BoNT-A (100 U) was well tolerated and resulted in significantly and clinically relevant improvements in all OAB symptoms, patient-reported benefits, and HR-QoL in patients inadequately managed by anticholinergic drugs [6,7,8,9]. However, there still exist substantial incidences of various treatment-related adverse events (AEs) (20%–43%) [10]. Acute urinary retention (AUR), large postvoid residual (PVR), difficulty in urination, and UTI are common AEs.

Interstitial cystitis/bladder pain syndrome (IC/BPS) is a clinical diagnosis based on symptoms including urinary frequency, urgency and bladder pain, pressure, and/or discomfort in the absence of other pathologic findings [11]. Although urothelial dysfunction, mast cell activation, neurogenic inflammation and C-fiber neuroplasticity are proposed to be the causes of IC/BPS [12], the pathogenesis of IC/BPS is not fully understood so far. The management of IC/BPS is mainly directed to amelioration of the bothersome symptoms such as bladder pain or urinary tract symptoms.

Intravesical botulinum toxin A has been suggested to be administered in IC/BPS patients if other treatments have not provided adequate symptom control and quality of life [13]. Recent studies have demonstrated the therapeutic effects of BoNT-A on the symptom relief of IC/BPS [14,15,16,17]. Common AEs included dysuria, the need for abdominal straining to void, large PVR, and the need for clean intermittent self-catheterization (CISC). However, the morbidity substantially reduced if the dose used was shifted from 200 to 100 U [13].

Although OAB and IC/BPS are different disease entities, intravesical BoNT-A injection was used to treat both syndromes in subjects with refractory conditions. In addition to telling the patients the advantage of the therapy, it is necessary to disclose the possible AEs before the procedures. The American Urological Association (AUA) guidelines for OAB and IC/BPS stated that the patients must be able and willing to perform, or accept the possibility of, CISC if BoNT-A injections are planned to be offered [13,18]. Considering the different nature of the diseases, it makes one curious to know if there any difference in the distribution of BoNT-A related AEs between the two groups of patients, which may be important in patient counseling before the treatment and might reflect the mechanisms of action of BoNT-A in the two syndromes. Thus, we conducted this study to compare AEs of intravesical BoNT-A injections between OAB and IC/BPS in women.

## 2. Results

### 2.1. Demographics

Eighty-nine women (aged 48.81 ± 11.81) with IC/BPS and 72 (aged 49.15 ± 10.85) with OAB were included in this study. The patient characteristics are listed in Table 1. At baseline, there was no significant difference in the mean age and most urodynamic parameters between the two groups. Exceptions were the significantly higher mean functional bladder capacity (FBC), day time frequency, nocturia, maximum flow rate (Qmax), and detrusor pressure at Qmax (Pdet) in the OAB group when compared with those in the IC/BPS group, showing the distinct nature of the two syndromes.

### 2.2. Effects of Botulinum Toxin A on Voiding Function

At six months after BoNT-A injections, the mean Overactive Bladder Symptom Score, urge incontinence episode, urgency episode and day time frequency decreased significantly in OAB patients. There was an overall successful rate of 51.4% (24 dry and 13 with increase of Patient’s Perception of Bladder Condition score ≥ 2) [19]. In the IC/BPS group, the mean O’Leary-Sant Symptom Index and Problem Index, pain visual analogue scale, daytime frequency and nocturia decreased and functional bladder capacity increased significantly at six months, making an overall successful rate of 46.1% (41 with a Global Response Assessment score ≥ 2) [20].

**Figure 1:**
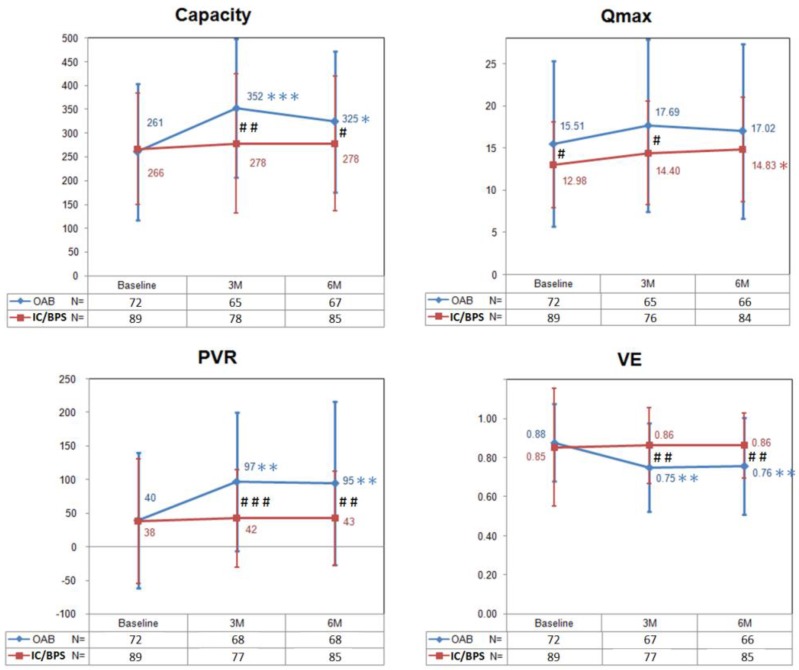
The changes of bladder capacity, maximum flow rate (Qmax), postvoid residual (PVR), and voiding efficiency (VE) in patients with overactive bladder (OAB) and interstitial cystitis/bladder pain syndrome (IC/BPS) after BoNT-A injections. Data are expressed as mean ± standard deviation. * *p* < 0.05, ** *p* < 0.01, *** *p* < 0.001 when compared with baseline data in each group. Paired *t* test was used. ^#^ *p* < 0.05, ^##^ *p* < 0.01, ^###^ *p* < 0.001 when compared between groups. Independent *t* test was used.

Figure 1 shows changes in the bladder capacity, Qmax, PVR, and voiding efficiency (VE) from baseline to six months after BoNT-A injections in OAB and IC/BPS patients. In the OAB group, the PVR and bladder capacity peaked and the VE decreased significantly at three months, and did not return to baseline level at six months. There was no significant change of Qmax within the six-month follow-up period. In the IC/BPS group, there was no change in the bladder capacity, PVR, and VE from baseline to six months after BoNT-A injections. Interestingly, the Qmax increased significantly at six months.

Taking these data together, there were significant differences in changes of capacity, Qmax, PVR and VE between the two groups (Figure 1). Patients in the OAB group had larger bladder capacity, PVR and lower VE at three and six months after BoNT-A injections when compared with those in the IC/BPS group. Comparing with the OAB group, the Qmax in the IC/BPS group was significantly lower at baseline but “recovered” at six months after BoNT-A injections.

### 2.3. Adverse Events after Botulinum Toxin A Injections

During the follow-up period, 58.3% of OAB women reported at least one AE, including gross hematuria (9.7%), UTI (27.8%), straining to void (8.3%), large PVR (>200 mL) (31.9%) and AUR (1.4%). On the other hand, 42.7% of IC/BPS patients found at least one AE, including UTI (6.7%), straining to void (30.3%) and large PVR (6.7%). There was no gross hematuria nor AUR experienced (Table 2). These data demonstrated the overall incidence of AEs was significantly higher in the OAB group than in the IC/BPS group. Patients in the OAB group suffered more frequently from events of hematuria, UTI, and large PVR but less frequently from event of straining to void than those in the IC/BPS group. There was no significant difference in events of AUR between the two groups.

## 3. Discussion

To our knowledge, this is the first study to compare the BoNT-A injection related AEs between OAB and IC/BPS patients. Our data demonstrated that by injecting 100 U of BoNT-A into the suburothelial space, the volume of bladder capacity and PVR increased, and the VE decreased significantly in women with OAB than those with IC/BPS within a six-month follow-up period. These results imply that the contractility of bladder in OAB patients might be more susceptible to BoNT-A injection than that in IC/BPS.

Although BoNT-A was applied to the treatment of both OAB and IC/BPS, the mechanisms of action may be different between these two syndromes because of their different pathophysiology. Intravesical injection of BoNT-A has been introduced to treat refractory IC/BPS since 2004 [21]. BoNT-A acts by cleaving the synaptosome-associated protein of 25 kd complex in the presynaptic terminal, which prevents formation of the SNARE system and then the release of neurotransmitter vesicles at the synaptic cleft. Consequently, the release of acetylcholine, calcitonin gene-related protein, substance-P and glutamate decreased and thus the nociceptive fiber discharge reduced [22], suggesting the therapeutic rationale of BoNT-A on IC/BPS. Furthermore, recent studies have demonstrated that BoNT-A has an anti-inflammatory effect on a cystitis rat model [23] and that injection of BoNT-A can reduce the production of nerve growth factor in the bladder resulting in satisfactory pain relief in IC/PBS patients [24,25].

In the last decade, intravesical injection of BoNT-A emerged as an effective treatment for OAB among patients refractory or intolerant to antimuscarinic agents [26]. BoNT-A significantly improves OAB symptoms and urodynamic parameters in OAB patients. The clinical efficacy of BoNT-A may be explained by its proposed dual mechanism of action in targeting both the afferent and efferent neuronal pathways of bladder control. BoNT-A can not only block the release of acetylcholine and other neurotransmitters from nerve fibers but also from urothelium. In addition, BoNT-A may decrease the expression of sensory receptors [27,28]. Since increased release of acetylcholine from neuronal and nonneuronal (urothelial) sources in the bladder during the storage phase was proposed to contribute to the pathophysiology of detrusor overactivity (DO) [29,30], which is the main target of BoNT-A injection, there is no wonder that the bladder contractility in OAB patients is more easily affected by BoNT-A injection than that in IC/BPS, resulting in higher volume of bladder capacity and PVR, and lower VE in OAB patients. This phenomenon suggests there might be different mechanisms of action of BoNT-A on the two syndromes, yet further investigations to compare the changes of sensory or motor proteins in the OAB and IC/BPS bladder before and after BoNT-A treatment are warranted.

Our data also disclosed that the Qmax in the IC/BPS group was significantly lower at baseline, but was raised to as high as in the OAB group at six months after BoNT-A injections. It has been well established that visceral pain syndromes, including irritable bowel syndrome, endometriosis, and IC/BPS, may be associated with non-relaxing pelvic floor muscles through central and peripheral sensitization and lowering of nociceptive thresholds, resulting in neuropathic up-regulation, hypersensitivity, allodynia and dysfunctional voiding [31]. Intravesical injection of BoNT-A may reduce the pain symptom and neurogenic inflammation inside the pelvis [23,24,25,32], and thus release the non-relaxing pelvic floor muscles and improve the dysfunctional voiding in IC/BPS patients.

When comparing the rates of AEs after BoNT-A injections between the two groups, we found that patients in the OAB group had higher incidences of hematuria, UTI, and large PVR, but lower incidence of straining to void than those in the IC/BPS group. Only one OAB and no IC/BPS patient experienced AUR. Although our result showed no significant difference in event of AUR between the two groups, 31.9% of OAB *vs.* 6.7% of IC/BPS women had large PVR (>200 mL), which is compatible with the change of voiding function after BoNT-A injections in both groups. Again, higher percentage of large PVR in the OAB group indicates its vulnerable nature to BoNT-A injections. In addition, UTIs have been reported in between 2% and 32% of OAB patients treated, and are usually associated with a large PVR volume [33], explaining the higher incidence of UTIs in OAB patients. It is difficult to clarify why OAB women experienced hematuria more frequently than IC/BPS. Since five of the seven (71%) OAB patients who had hematuria also experienced UTIs, the finding that the OAB group had higher incidence of hematuria may partly be a confounding result of UTIs. Moreover, chronic inflammation resulting in more advanced fibrosis in the suburothelial space in IC/BPS patients may account for these findings in part.

Since the FDA-approved dose of BoNT-A for OAB treatment is 100 U, some AEs appear to occur less frequently in comparison with previous studies using higher doses [18]. For example, rates of urinary retention have decreased from 43% to <20%. Similarly, the percent of patients requiring CISC have decreased from 43% to less than 10%. However, recent studies using 100 U of BoNT-A to treat IC/BPS revealed a much lower percentage (0%–2%) of patients either suffering from urine retention or needing CISC [15,17,34,35]. The most common AE appeared to be dysuria. The current study also demonstrated that 30.3% of IC/BPS patients experienced straining to void. Only 6.7% had large PVR and none had urine retention, suggesting that the bladder contractility in IC/BPS patients seems being less interfered by BoNT-A injection. Although the AUA guideline statement addressing the possibility of CISC post-treatment in IC/BPS patients cannot be defeated at present [13], the very low incidence of urine retention and need for CISC may encourage the physicians and patients to receive BoNT-A injections, and help in the counseling before treatment.

One may criticize the use of hydrodistention (HD) in IC/BPS patients to be the major weakness in the comparison of AE between the two disorders in this study. BoNT-A injections followed by HD has been proved to be more effective than HD alone in treating IC/BPS in our previous prospective, randomized study [16]. In order to achieve the optimal therapeutic effect, we routinely use this method to treat our IC/BPS patients. However, the above study also demonstrated that HD alone could only improve the subjective parameters (IC symptom scores) but not objective (voiding diary or urodynamic) parameters and that the AE caused by HD was minimal (only one kind of AE, dysuria, presented in one out of 23 patients). Hence, the influence of HD on the comparison of voiding function and AEs after BoNT-A injections between OAB and IC/BPS women in this study is expected to be very limited.

Other limitations of this study are data from single center, nonconsecutive enrollment of patients, and a lack of comparing comorbidities and its retrospective nature. Nevertheless, the male patients have been excluded and the patients’ ages have been adjusted between the two groups to minimize the confounding risk factors for voiding dysfunction.

## 4. Materials and Methods

### 4.1. Patient Enrollment

In this retrospective study, female patients with IC/BPS were retrieved from a database containing one hundred four subjects who have undergone intravesical BoNT-A injection followed by hydrodistention from 2005 to 2013. They had been treated with oral pentosan polysulfate sodium, intravesical instillation of heparin, hyaluronic acid, or oral tricyclic antidepressant for at least 6 months, but the symptoms remained unchanged or relapsed. A diagnosis of IC/BPS has been established based on characteristic symptoms and cystoscopic findings of glomerulation, petechia, or mucosal ulceration [36].

**Figure 2:**
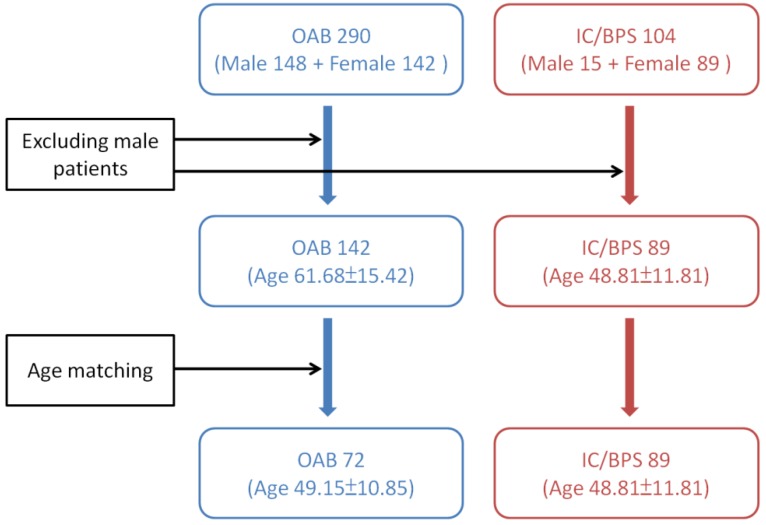
Instruction of patient selection from the databases of overactive bladder (OAB) and interstitial cystitis/bladder pain syndrome (IC/BPS).

Previous studies have revealed that male gender, age > 61 years old, those with a baseline Qmax ≤ 15  mL/sec, PVR ≥ 100 mL, and VE < 90% are risk factors of urine retention developed after BoNT-A injections in OAB patients [10,37]. Since patients with OAB are generally older and consisting of a higher percentage of males than those with IC/BPS, we excluded the male patients in both study groups. And only age-matching females with OAB were selected from a database consisting of 290 idiopathic DO subjects for comparing the effects of BoNT-A injection on voiding function and AEs. They have received intravesical BoNT-A injections from 2005 to 2014. These patients had undergone life style modification therapy and treatment with at least two antimuscarinic agents for more than 3 months, but they were still bothered by severe urgency (defined as urgency severity score of 3) or urge urinary incontinence, resulting in a low quality of life. The detailed instruction for patient selection from the two databases is shown in Figure 2.

### 4.2. Botulinum Toxin Injections

All the patients received intravesical suburothelial injections of a total of 100 U of BoNT-A at 20 to 40 sites in the bladder body, excluding the trigone. In IC/BPS patients, cystoscopic hydrodistention was performed to an intravesical pressure of 80 cm water for 15 min immediately after the injections. The detailed technique for suburothelial injection was described in previous reports [32,38].

### 4.3. Patient Follow-Up

Overactive Bladder Symptom Score for the OAB group, O’Leary-Sant Symptom and Problem Index and pain visual analog scale for the IC/BPS group, and 3-day voiding diary were recorded at baseline and every follow-up time point. Videourodynamic study was routinely performed at baseline. The cystometric bladder capacity, Pdet, Qmax, and PVR were recorded. During the follow-up period, uroflowmetry for Qmax, voided volume, and PVR were measured at each visit. The total bladder capacity was calculated by summation of the voided volume and PVR. The VE was calculated as the ratio of voided volume of the total bladder capacity. The total bladder capacity, Qmax, PVR and VE were used to analyze the occurrence of AEs within 6 months after BoNT-A treatment.

All patients were closely monitored at 2 weeks, 1 month, 3 months, and every month thereafter until the response to BoNT-A disappeared. Procedure-related AEs were recorded during the 6-month follow-up period after BoNT-A treatment. AEs included AUR (patients had severe difficulty in urination with PVR > 350 mL and an indwelling catheter or CISC was necessary); gross hematuria; large PVR (>200 mL); straining to void (feeling difficulty urinating and needing abdominal straining to empty the bladder, which was not experienced prior to treatment); and UTIs (symptomatic or asymptomatic with white blood cell count > 10/high power field in urinalysis).

### 4.4. Statistical Analysis

The changes in total bladder capacity, Qmax, PVR and VE and the incidences of AEs during the follow-up period were analyzed and compared between patients in OAB and IC/BPS groups. Statistical comparisons between the groups were tested using a chi-square test for categorical variables, and an independent t or paired *t* test for continuous variables. Statistical analyses were performed using SPSS 18.0 statistical software (SPSS Inc., Chicago, IL, USA).

## 5. Conclusions

Patients in the OAB group had higher PVR volume and lower VE than those in the IC/BPS group after BoNT-A treatment. These results imply that the bladder contractility of OAB women might be more susceptible to BoNT-A, which might reflect the different mechanisms of action of BoNT-A on bladder dysfunction. Further investigations to compare the changes of sensory or motor proteins in the OAB and IC/BPS bladder at baseline and after BoNT-A treatment might provide evidence for this speculation.

## Figures and Tables

**Table 1:** Characteristics of study patients.

Group	OAB (*N* = 72)	IC/BPS (*N* = 89)	*p*
Age (years)	49.15 ± 10.85	48.81 ± 11.81	0.777
Functional bladder capacity (mL)	351.43 ± 135.21	124.72 ± 76.91	0.000 *
Daytime frequency (times/day)	34.05 ± 14.56	15.64 ± 7.88	0.000 *
Nocturia (times/night)	8.05 ± 2.99	4.90 ± 4.93	0.009 *
Urgency (times/24 h)	33.00 ± 17.87	-	-
Urge urinary incontinence (times/24 h)	10.57 ± 12.98	-	-
Visual analogue scale	-	5.43 ± 2.24	-
Maximum flow rate (Qmax) (mL/s)	15.73 ± 9.69	12.62 ± 5.48	0.012 *
Voided volume (mL)	224.86 ± 122.24	244.41 ± 112.12	0.364
Postvoid residual (mL)	39.19 ± 100.28	38.01 ± 93.26	0.916
Total bladder capacity (mL)	260.93 ± 143.25	266.19 ± 117.15	0.800
Voiding efficiency	0.88 ± 0.20	0.85 ± 0.30	0.577
First sensation of filling (mL)	112.14 ± 68.90	117.33 ± 53.28	0.744
Strong desire to void (mL)	210.86 ± 120.41	197.45 ± 87.74	0.597
Cystometric bladder capacity (mL)	264.61 ± 145.28	274.72 ± 109.92	0.714
Detrusor pressure at Qmax (cm H_2_O)	27.49 ± 13.60	19.19 ± 10.65	0.000 *

* *p* < 0.05. Independent *t* test.

**Table 2:** The adverse events in patients with overactive bladder and interstitial cystitis/bladder pain syndrome.

Adverse Events	OAB (%)	IC/BPS (%)	*p*
Hematuria	7 (9.7)	0 (0)	0.003 ^a^
UTI	20 (27. 8)	6 (6.7)	0.000 ^a^
Straining to void	6 (8.3)	27 (30.3)	0.001 ^a^
PVR > 200 mL	23 (31.9)	6 (6.7)	0.000 ^a^
AUR	1 (1.4)	0 (0)	0.265
Any	42 (58.3)	38 (42.7)	0.048 ^b^

^a^ Pearson chi-square with Fisher exact correction; ^b^ Pearson chi-square test. UTI: urinary tract infection. PVR: postvoid residual. AUR: acute urinary retention.

## Abbreviations

The following abbreviations are used in this manuscript:

OAB: overactive bladder
UTI: urinary tract infection
BoNT-A: onabotulinumtoxinA
HR-QoL: health-related quality of life
AE: adverse event
AUR: acute urinary retention
PVR: postvoid residual
IC/BPS: interstitial cystitis/bladder pain syndrome
CISC: clean intermittent self-catheterization
AUA: American Urological Association
FBC: functional bladder capacity
Qmax: maximum flow rate
Pdet: detrusor pressure at Qmax
VE: voiding efficiency
DO: detrusor overactivity
HD: hydrodistention
